# Template-mediated Synthesis of Hollow Microporous Organic Nanorods with Tunable Aspect Ratio

**DOI:** 10.1038/srep31359

**Published:** 2016-08-10

**Authors:** Qingyin Li, Shangbin Jin, Bien Tan

**Affiliations:** 1 School of Chemistry and Chemical Engineering, Huazhong University of Science and Technology, Wuhan, 430074, China

## Abstract

Hollow microporous organic nanorods (HMORs) with hypercrosslinked polymer (HCPs) shells were synthesized through emulsion polymerization followed by hypercrosslinking. The HMORs have tunable aspect ratios, high BET surface areas and monodispersed morphologies, showing good performance in gas adsorpion.

During the past decades, microporous organic polymers (MOPs) with tunable morphology, pore structure and high stability have attracted considerable attentions because of their potential applications in many areas, such as gas adsorption^1^,^2^,^3^, catalysis^4^,^5^,^6^,^7^, sensor^8^,^9^, energy storage^10^ and pollutant removal^11^,^12^. Most of the researches have focused on either developing new synthetic methodologies or new functionalities. However, recently there are some efforts on the morphology control of MOPs which is also important, such as quasi-zero-dimensional (0-D) microspheres^13^, and one-dimensional (1-D) nanotubes^14^, two-dimensional (2-D) film structures^15^, as well as three-dimensional (3-D) monoliths^16^. The hollow microporous capsules (HMOCs) combine the advantages of micropores in the shells and hollow cores. The microporous shells render such capsules to possess high surface areas and large pore volumes, while the hollow cores enable the guest materials to stay inside and confine them into nanostructural environments. Therefore, MOPs with hollow structures are interesting materials with potential applications in drug release^17^, catalysis^4^,^5^,^6^,^7^, oil removal^12^ and template for inorganic materials^18^,^19^ or precursor material for porous carbon^20^,^21^,^22^. The most studied HMOCs are zero-dimensional (0-D) whereas one-dimensional (1-D) HMOCs have received a little attention recently^19^,^23^.

Intrinsic anisotropy of rod-like nanostructures renders them attractive for a number of applications including those in electrorheological fluid^24^, catalyst^25^,^26^, conducting polymer^27^, Pickering emulsifier^28^. Compared with 0-D particles, high aspect ratio (AR) of 1-D material makes it have a higher active surface area per unit substrate surface area, higher packing densities of microstructural elements, higher throughput in continues flow systems, which make rod-like nonmaterial display better performance in certain fields. For example, worm-like and rod-like block copolymer nanoparticles could be taken up better by the cells than that of 0-D block copolymer nanoparticles^29^. Polyaniline coated mesoporous silica nanorods (AR = 5 and 10) are preferable to sphere one (AR = 1) in electrorheological activity^30^. Relative to traditional spherical fillers, rod-like fillers can increase the bulk conductivity of conducting polymer nanocomposites at very low filler concentrations^31^. Tuning the AR of rod-like nanomaterials has significant implications of their properties and thus the potential applications. For example, Wang *et al*. demonstrated that gold nanoshells with a moderate AR of about 2 could result in the most effective elimination of murine melanomas^32^. Li *et al*. demonstrated that the active molecules release faster from longer SiO_2_/Polymer hybrid capsule (AR = 7) than from shorter ones (AR = 4) under similar conditions^33^.

Hypercrosslinked polymers (HCPs) are important MOPs, which can be readily prepared with high surface area and easily scaled up. We have recently reported a method to fabricate 0-D HMOCs with HCPs shells. The HMOCs show high efficiency for drug loading^17^ and can also be used as support for platinum nanoparticles, which show excellent catalytic properties in hydrogenation reactions^4^. Despite of achievement in HMOCs, nanorods-like hollow microporous organic polymers with HCPs shell have not yet been reported.

We report the template synthesis of hollow microporous nanorods (HMORs) with tunable AR by using silica as template and HCPs as hollow shells for the first time (Fig. 1). Tunable AR rod-like silica nanoparticles were prepared following the reported method^34^. The as-made SiO_2_ nanorods were modified by using 3-(trimethoxysilyl)propyl methacrylate (MPS) to obtain m-SiO_2_. Then emulsion polymerization was carried out by using m-SiO_2_ (0.6 g) as template; the mixture of styrene (St, 7.5 mL) and 10 v% divinylbenzene (DVB, 0.94 mL) as monomer to produce core-shell SiO_2_@PS-DVB. Using formaldehyde dimethyl acetal (FDA, 1.71 mL for 1 g SiO_2_@PS-DVB) as external crosslinker and 3.14 g FeCl_3_ as catalyst, aromatic ring of PS-DVB shell was crosslinked by Friedel-Crafts reaction. As a result, rod-like product with HCPs shell (rod-HCPs) was formed. Lastly, SiO_2_ in rod-HCPs was etched by HF to form HMORs. By using SiO_2_ nanorods with AR of 3.4 and 7.3 as template, the corresponding organic nanorods, i.e., HMORs-3 and HMORs-7 were obtained respectively.

## Result and Discussion

The formation of SiO_2_-3, m-SiO_2_-3, SiO_2_@PS-DVB-3, rod-HCPs-3 and HMORs-3 was studied by Fourier transform infrared spectroscopy (FT-IR) measurements (Fig. S1, ESI). The peak at 1710 cm^−1^ of m-SiO_2_-3 can be ascribed to the vibration of the C = O bond of MPS (compared with SiO_2_), which verifies the successful modification of MPS. FT-IR spectra of SiO_2_@PS-DVB-3 indicates the successful surface modification of PS-DVB, as evident from the characteristic peak of phenyl (=C-H) at 3030–3080 cm^−1^, overtones of phenyl at 1670 cm^−1^, peak of phenyl (C = C) at 1600 cm^−1^, 1500 cm^−1^and 1460 cm^−1^ and peak of phenyl (=C-H) at 810 cm^−1^, 750 cm^−1^ and 690 cm^−1^. The FT-IR spectra of the rod-HCPs-3 shows almost a complete disappearance of peak of phenyl (=C-H) at 3030–3080 cm^−1^, which is indicative of a low conversion of =C-H after hypercrosslinking. For HMORs-3, the band of Si-O-Si groups disappears at 1100 cm^−1^.

SEM and TEM images of m-SiO_2_ show bullets-like morphology (Fig. 2a,c,e,g). The core-shell structure has different morphologies: a layer of PS-DVB is deposited on m-SiO_2_ surface and the morphology has no difference after hypercrosslinking (Fig. S2, ESI). After HF treatment, HMORs-3 and HMORs-7 are both monodispersed and remain completely (Figs 2b,d and S3, ESI), which are the same as rod-HCPs. TEM images show that after removing the template the inner hollow cavity of HMORs-3 and HMORs-7 are clearly formed and the shape of silica template is almost retained (Fig. 2f,h).

The average length, diameter and AR are calculated based on 100 rods (Fig. 2 and Table S1, ESI). The average AR (calculated by the length/diameter (L/D) of the rods) is about 3.4 for m-SiO_2_-3, which has an average length of 1.1 μm and average width of 0.3 μm. The average length, width and average AR is 1.2 μm, 0.5 μm and 2.6 respectively for HMORs-3. The average thickness of the shell is about 70 nm. The average AR of HMORs-7 decreases from 7.3 to 5.6 and the average length and diameter increase from 2.0 μm and 0.3 μm to 2.1 μm and 0.4 μm respectively. The shell thickness of HMORs-7 is about 50 nm.

**Figure 1:**
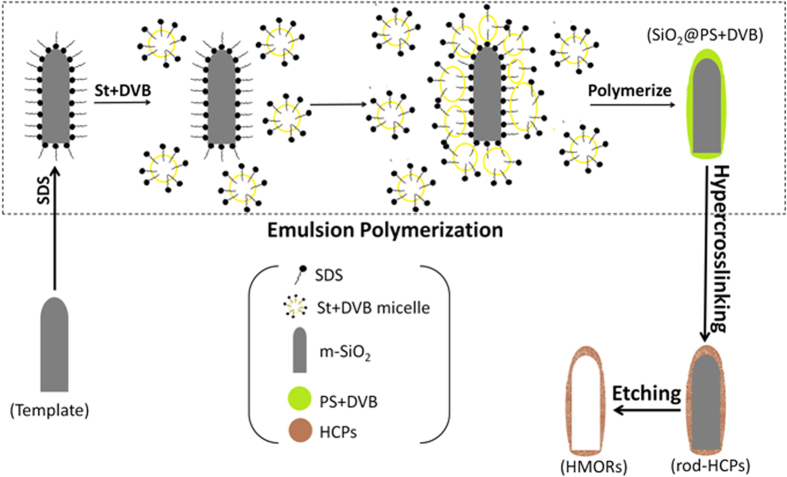
Schematic description of synthetic route of HMORs.

**Figure 2:**
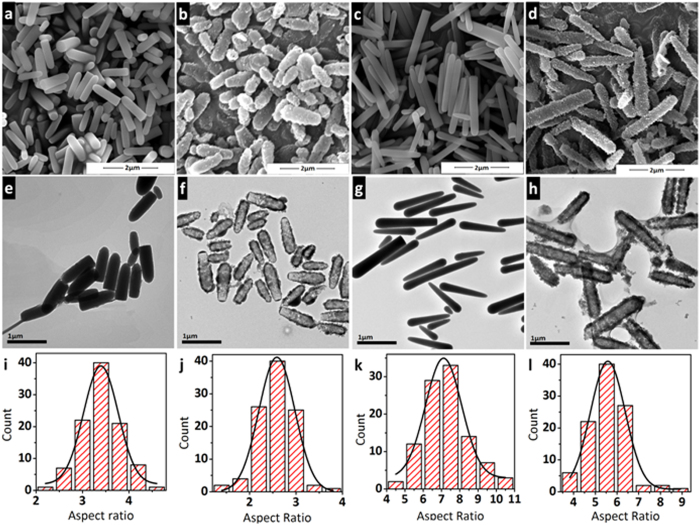
SEM images of **m-SiO2-3 (a)**, **HMORs-3 (b)**, **m-SiO2-7 (c)** and **HMORs-7 (d)**, TEM images of **m-SiO2-3 (e)**, **HMORs-3 (f)**, **m-SiO2-7 (g)** and **HMORs-7 (h)** and AR distribution of **m-SiO2-3 (i)**, **HMORs-3 (j)**, **m-SiO2-7 (k)** and **HMORs-7 (l)** (The average AR was calculated by AR found by each particle).

A previous study has shown that spherical HMOCs have smooth shells^17^. However, HMORs-3 and HMORs-7 both have rough shells with some small particles adhered to them (Figs 2 and S4, ESI). We presume that the mechanism of HMORs formation may be different from that of sphere (Fig. 1). We propose that SDS surfactant forms a layer on the surface of each m-SiO_2_ in water. When St-DVB mixture is added to the system, micelles are formed, which contact with the hydrophobic surfaces of m-SiO_2_ due to the presence of SDS layer on the surfaces and subsequently get polymerised. However, the length of the template makes it difficult for one micelle to wrap around it completely. So there are many micelles adhered to one rod and, as a result, the shell has varying thickness along with the formation of smaller PS-DVB particles. The longer templates are even less likely to be wrapped uniformly by the micelles, which might have resulted in the formation of HMORs with non-uniform shell thickness. HMORs-7 may easily break under some harsh conditions, which agree well with the SEM and TEM images.

Table 1 summarizes the surface areas and the pore volumes of HMORs-3 and HMORs-7. The surface areas are calculated using Brunauer−Emmett−Teller (BET) and Langmuir methods respectively. Both m-SiO_2_-3 and SiO_2_@PS-DVB-3 have very low BET surface area (9 m^2^g^−1^ and 10 m^2^g^−1^). In contrast, rod-HCPs-3 has a BET surface area as high as 395 m^2^g^−1^, which increased sharply due to the formation of micropores by hypercrosslinking in the shells. After removing the less porous silica template, the surface area of HMORs-3 (527 m^2^g^−1^) is further increased. From BET results, we can calculate that HCPs shell contributes about 75% of BET surface area to the rod-HCPs-3, which means that the weight percentage of HCPs shell is 75%. Both m-SiO_2_-3 and SiO_2_@PS-DVB-3 only have very small pore volume (0.01 and 0.02 cm^3^g^−1^). Rod-HCPs-3 and HMORs-3 have much higher pore volumes of 0.62 and 0.75 cm^3^g^−1^ respectively. All the results from HMORs-7 are almost the same as HMORs-3. To our surprise, the porosity of HMOCs^17^ and HMORs are almost the same. However, HMORs have higher pore volume as compared with HMOCs, which may be due to the presence of more macro- and mesopores in HMORs.

**Figure 3:**
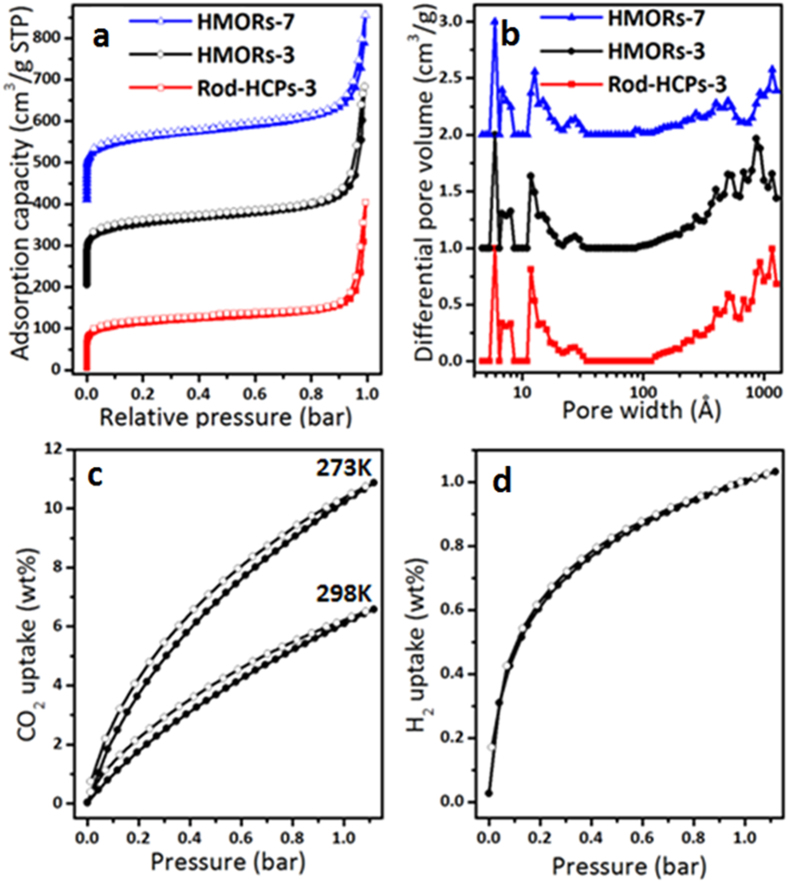
N_2_ sorption isotherms of **rod-HCPs-3**, **HMORs-3** and **HMORs-7** at 77 K (**a**, the isotherms of **HMORs-3** and **HMORs-7** were shifted vertically by 200, 400 cm^3^g^−1^, respectively), pore distribution of pore size of **rod-HCPs-3**, **HMORs-3** and **HMORs-7** calculated using DFT methods (**b**, the isotherms of **HMORs-3** and **HMORs-7** were shifted vertically by 1, 2 cm^3^g^−1^, respectively). CO_2_ adsorption isotherm at 273 K and 298 K (**c**) and H_2_ adsorption isotherm at 77 K (**d**) of **HMORs-3**.

The analysis of N_2_ sorption isotherms shows the existence of hierarchical pores in HMORs-3 and HMORs-7 (Fig. 3a). The adsorption isotherms show a steep nitrogen gas uptake at lower relative pressure (P/P_0_ < 0.001) indicating abundant micropore structures, a slight hysteresis loop reflecting a spot of the mesopores, and a sharp rise at medium and high pressure region (P/P_0_ = 0.8–1.0) indicating the presence of macropores in these materials. The pore size distributions also confirm the presence of such heterogeneous porous structures (Fig. 3b). The porosities of HMORs-3 and rod-HCPs-3 are almost the same as some difference in response. Gas storage is one of the most promising applications for porous materials. The CO_2_ uptake for HMORs-3 is 10.87 wt% at 273 K and 6.58 wt% at 298 K, 1 bar (Fig. 3c). The H_2_ storage capacity for HMORs-3 is 1.03 wt% at 77 K, 1 bar (Fig. 3d). The CO_2_ uptake capacity of HMORs-3 is comparable to that of many reported MOPs with higher surface areas, such as diamino conjugated microporous polymers (DA-CMP-1, 10.78 wt% at 273 K and 1.13 bar, 662 cm^3^g^−1^ and DA-CMP-2, 7.22 wt% at 273 K and 1.13 bar, 603 cm^3^g^−1^)^1^, hybrid porous polymers (HPP-3, 2.73 wt%, at 298 K and 1 bar, 904 cm^3^g^−1^)^3^ and homogeneous CMP aerogels (PTEB-F-10, 9.15 wt% at 273 K and 1.13 bar, 965 cm^3^g^−1^)^35^.

**Figure 4:**
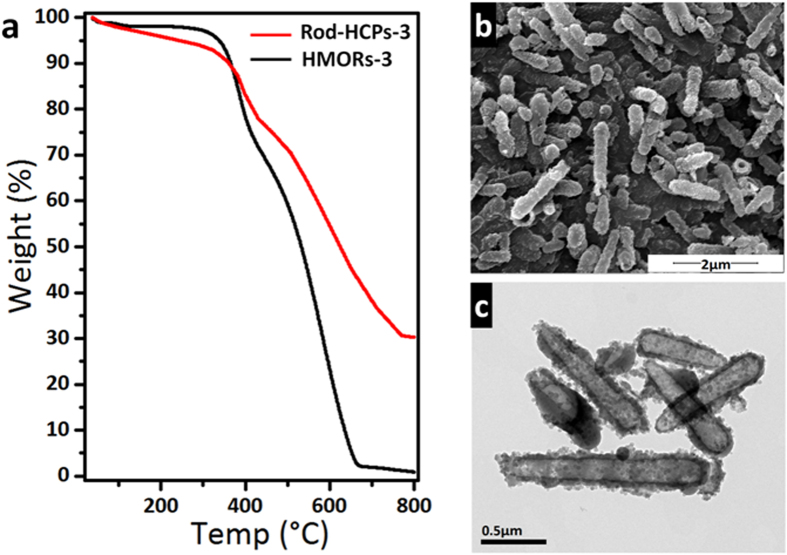
TGA **rod-HCPs-3** of and **HMORs-3** (**a**), SEM (**b**) and TEM (**c**) image of **C-HMORs-3.**

The thermal stability of the HMORs-3 was studied by thermal gravimetric analysis (TGA) as shown in Fig. 4a. Both HMORs-3 and rod-HCPs-3 began to decompose at about 400 °C. HCPs, however, has a slightly higher thermal stability because of higher thermal resistance of SiO_2_. We can see from the result, HMORs undergo degradation in two steps, which are attributed to primary unzipping or depropogation processes of polymer chains and the secondary decomposition reactions at higher temperatures. The primary degradation is happened at around 450 °C and this process undergoes further at higher temperatures in the second step completed by about 650 °C^36^. It is shown that the weight loss of polymer is 72%, which agrees well with the results calculated from BET surface area (75%). HMORs-3 was further heated in a tubular furnace to 700 °C at a rate of 5 °C min^−1^ under N_2_ protection (C-HMORs-3). As indicated by SEM and TEM images (Fig. 4b,c), some HMORs-3 break after heat treatment but still keep their rod-like morphology instead of breaking into fragments. BET surface area of C-HMORs-3 is still retained as high as 444 m^2^g^−1^. As meso/micro-porous materials and carbon nanotubes have played important role in improving the electrochemical properties, HMORs may be a potential carbon precursor to produce similar materials^20^,^21^,^22^,^37^,^38^.

**Figure 5:**
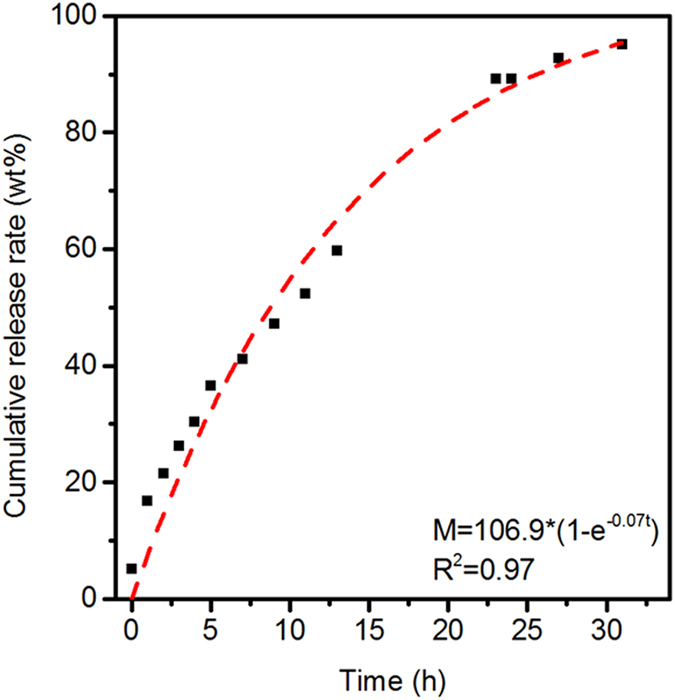
Drug release profile of HMORs-3. Red line is fitting line.

HMOCs have been confirmed nontoxic in pervious study^17^. As rod-like particles could be taken up better by the cells^29^. HMORs were also evaluated for their possible potential applications in drug release. The drug loading efficiency of HMORs-3 was calculated from the data obtained with UV-vis spectrophotometer. HMORs-3 can uptake 1.54 g/g (ibuprofen/HMORs). Drug release kinetics of HMORs-3 fit for the first order model which indicates that the release mechanism of ibuprofen is mainly controlled by the simple diffusion (Fig. 5).

## Conclusion

In summary, we have successfully demonstrated the synthesis of rod-like HMORs with tuneable AR and HCPs shells, for the first time, which have high surface areas and pore volumes. The formation of rod-like morphologies is clearly revealed by SEM and TEM observations. HMORs have decent and comparable performance in H_2_ and CO_2_ adsorption and storage. Moreover, the HMORs possessing first order drug release kinetics, which indicate their attractive applications in medical field. This research paves the way for the development of such HCPs with more advanced and controlled morphologies, which will be beneficial for applications in separation technologies, drug release as well as other fields in future.

## Method

### Preparation of SiO_2_ template

Rod-like silica was fabricated using a reported method^34^. To get different SiO_2_ with different aspect ratio, different amount of NH_3_·H_2_O was added (from 4 to 12 mL). The more NH_3_·H_2_O was added the less aspect ratio was obtained.

The SiO_2_ rods were modified by using MPS (m-SiO_2_): a mixture of MPS and TEOS (1 mL:1 mL) was dropped into the dispersion of SiO_2_ rod in 100 mL, 12 mL NH_3_·H_2_O and 10 mL DI water.

### Preparation of SiO_2_@PS-DVB nanoparticles

The SiO_2_@PS-DVB prececers were obtained by emulsion polymerization. Briefly, SDS (0.4 g) and NaHCO_3_ (0.24 g) were dissolved in distilled water (100 mL) then added the dispersion of m-SiO_2_ (0.6 g) in ethanol (10 mL). After adding the styrene (7.5 mL), 10% (v %) DVB (0.94 mL) and K_2_S_2_O_8_ (0.1 g), the emulsion polymerization was heated at 85 °C under inert gas protection for 2 h. The emulsion of SiO_2_@PS-DVB was centrifuged (12000 g) for 15 min and washed by water and ethanol then dried in vacuum oven to get SiO_2_@PS-DVB rod.

### Preparation of HMORs

The SiO_2_@PS-DVB (1.0 g) was swollen in DCE (20 mL) about 1 h. FDA (1.73 mL) was added to the mixture and then added FeCl_3_ (3.11 g). The Friedel-Crafts-type hypercrosslinking reaction was stirred at 45 °C for 5 h to form original network, then heated at 80 °C for 19 h. The resulting microporous nanoparticles (rod-HCPs) were filtered and washed followed washed with methanol in a Soxhlet for 24 h, and used HF to etch the silica core, finally dried in vacuum oven at 60 °C for 24 h. The brown HMOCs were obtained.

### Characterization of materials

The size of particles was measured from the scanning electron microscopy (FE-SEM, FEI Sirion 200 field emission scanningelectron microscope) images. The product powders were mounted on aluminum studs using adhesive graphite tape and sputter-coated with platinum before analysis. The average particle length and diameter calculated using ImageJ software. Gas adsorption/desorption analysis were carried out volumetrically using a Micromeritics ASAP 2020 M surface area and porosity analyzer (Micromeritics, USA). A liquid nitrogen bath (77 K) was used for the nitrogen and hydrogen isotherms, and an ethanol bath (273 K and 298 K) was used for the carbon dioxide isotherm. The purity of the gases used was 99.999% for nitrogen, hydrogen and 99.99% for carbon dioxide. Prior to the measurements, samples (0.1–0.2 g) in the analysis chamber were subjected to a vacuum of 10^−5^ bar at 110 °C for 8 h. The surface areas were calculated from nitrogen adsorption data by Brunauer–Emmett–Teller (BET) or Langmuir analysis. Pore size distributions were derived from the adsorption branches of the isotherms using the Tarazona nonlocal density functional theory (NLDFT) model assuming slit pore geometry; the NLDFT software is an integral of the ASAP 2020 M instrument. Total pore volumes were evaluated from nitrogen adsorption/desorption isotherms at relative pressure P/P_0_ = 0.99. Thermogravimetric (TG) analyses were carried out under a heating rate at 10 °C·min^−1^ in air, using a PerkinElmer Diamond TG/DTA. Fourier transform infrared (FT-IR) spectra were recorded under ambient conditions in the wavenumber range of 4000–400 cm^−1^ using a Bruker VERTEX 70 FT-IR Spectrometer (Bruker, Germany). Transmission electron microscopy (TEM) images were taken using a Tecnai G2 T20 U-TWIN HRTEM (FEI Corp., USA) instrument.

### Drug loading and release

A typical procedure for the loading of ibuprofen in HMOCs-3 is as follows: 150 mg of HMOCs-3 was suspended in 5 ml of 90 mg/ml ibuprofen solution in hexane under stirring for 96 h in a closed container to avoid the evaporation of hexane. The drug-loaded sample was then separated from the solution by vacuum filtration, washed with hexane, and dried at room temperature. The drug-loaded samples (200 mg) were then transferred to dialysis bag, and the release rate was obtained by soaking the drug-loaded samples in 100 ml of simulated body fluid (PBS, pH = 7.4, buffer solution, 37 °C) at pre-determined time intervals, 2 ml samples were withdrawn periodically for analysis and the remaining suspension replenished with an equal volume i.e., 3 mL of PBS immediately. Samples were analyzed for ibuprofen content at 264 nm using UV-Vis spectrophometer.

## Additional Information

**How to cite this article**: Li, Q. *et al*. Template-mediated Synthesis of Hollow Microporous Organic Nanorods with Tunable Aspect Ratio. *Sci. Rep*. **6**, 31359; doi: 10.1038/srep31359 (2016).

## Supplementary Material

Supplementary Information

## Figures and Tables

**Table 1:** Surface area and porosity of HMORs.

Sample	S_BET_^[a]^ m^2^g^−1^	S_L_^[b]^ m^2^ g^−1^	M.A.^[c]^ m^2^g^−1^	PV^[d]^ cm^3^g^−1^	M.P.V.^[e]^ cm^3^g^−1^
**m-SiO**_**2**_**-3**	9	12	—	0.01	—
**SiO**_**2**_**@PS-DVB-3**	10	15	—	0.02	—
**rod-HCPs-3**	395	527	226	0.62	0.10
**HMORs-3**	527	704	297	0.75	0.13
**HMORs-7**	552	738	281	0.70	0.13
**10%-HMOCs**^9^	516	691	351	0.35	0.16

^[a]^Surface area calculated from nitrogen adsorption isotherms at 77.3 K using BET equation. ^[b]^Surface area calculated from nitrogen adsorption isotherms at 77.3 K using Langmuir equation. ^[c]^t-Plot micropore area. ^[d]^Pore volume calculated from nitrogen isotherm at P/P_0_ = 0.995, 77.3 K. ^[e]^t-Plot micropore volume.
