# Supplementary material for: Template-mediated Synthesis of Hollow Microporous Organic Nanorods with Tunable Aspect Ratio
Source: Sci Rep. 2016 Aug 10;6:31359. doi: 10.1038/srep31359 (PMC4979212; doi:10.1038/srep31359)
Supplement: Supplementary Information [file srep31359-s1.pdf]

Supporting Information

Template-mediated Synthesis of Hollow Microporous Organic Nanorods with Tunable Aspect Ratio

Qingyin Li[1], Shangbin Jin[1], Bien Tan* [1]

[1] School of Chemistry and Chemical Engineering, Huazhong University of Science and Technology, Wuhan, 430074, China.

* E-mail: bien.tan@mail. hust.edu.cn; Fax: +86 27 87543632; Tel: +86 27 87558172.

Experimental

Materials

Anhydrous ferric chloride ($FeCl_3$), tetraethylorthosilicate (TEOS), ammonia water ($NH_3 \cdot H_2O$), 1,2-dichloroethane (DCE), styrene (St), methyl methacrylate (MMA), $NaHCO_3$, sodium dodecyl sulfate (SDS), polyvinylpyrrolidone K-30 (PVP), trisodium citrate dehydrate, methanol and absolute ethanol were analysis grade and purchased from Sinopharm Chemical Reagent Co., Ltd. Divinylbenzene (DVB, Aldrich, 80 %), 1-pentanol (Aladdin, 98 %) and formaldehyde dimethyl acetal (FDA, Aladdin, 98 %) were used as received. Potassium persulfate ($K_2S_2O_8$, Fisher) was recrystallized from deionized water prior to drying under reduced pressure. 3-(trimethoxysilyl)propyl methacrylate (MPS) and other reagents of analytical grade were utilized without further purification.

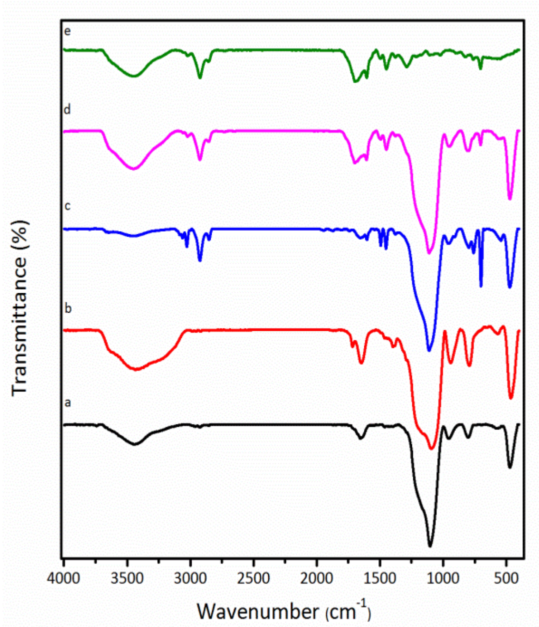

Fig. S1 FT-IR spectra of **SiO_2-3** (a), **m-SiO_2-3** (b), **SiO_2@PS-DVB-3** (c), **rod-HCPs-3** (d) and **HMORs-3** (e).

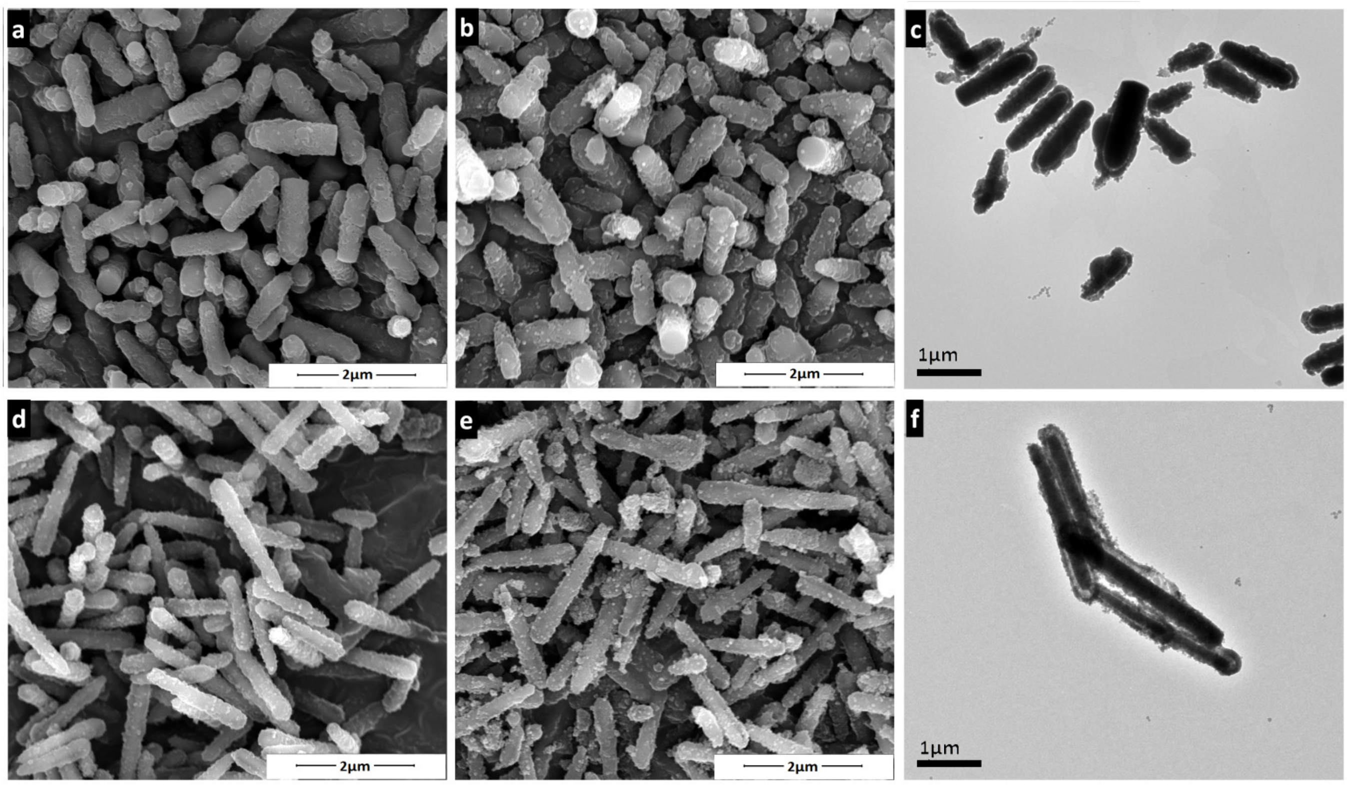

Fig. S2 SEM image of **SiO_2@PS-DVB-3** (a), **rod-HCPs-3** (b), **SiO_2@PS-DVB-7** (d), **rod-HCPs-7** (e), TEM image of **rod-HCPs-3** (c) and **rod-HCPs-7** (f)

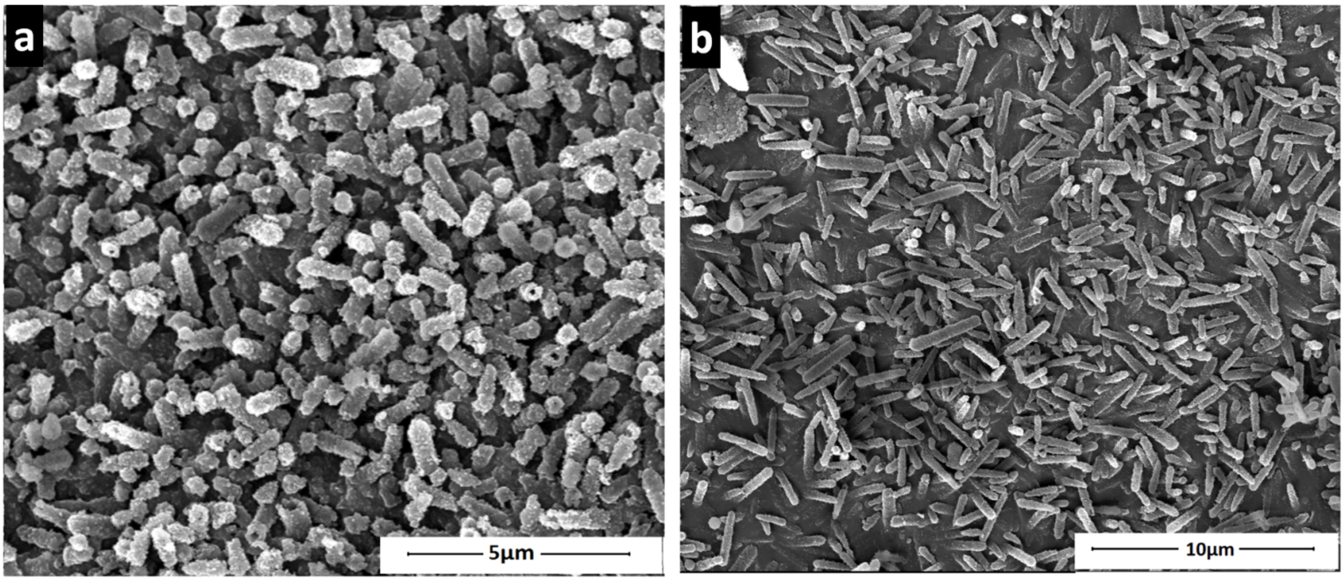

Fig. S3 SEM image of **HMORs-3** (a, 20,000×) and **HMORs-7** (b, 10,000×)

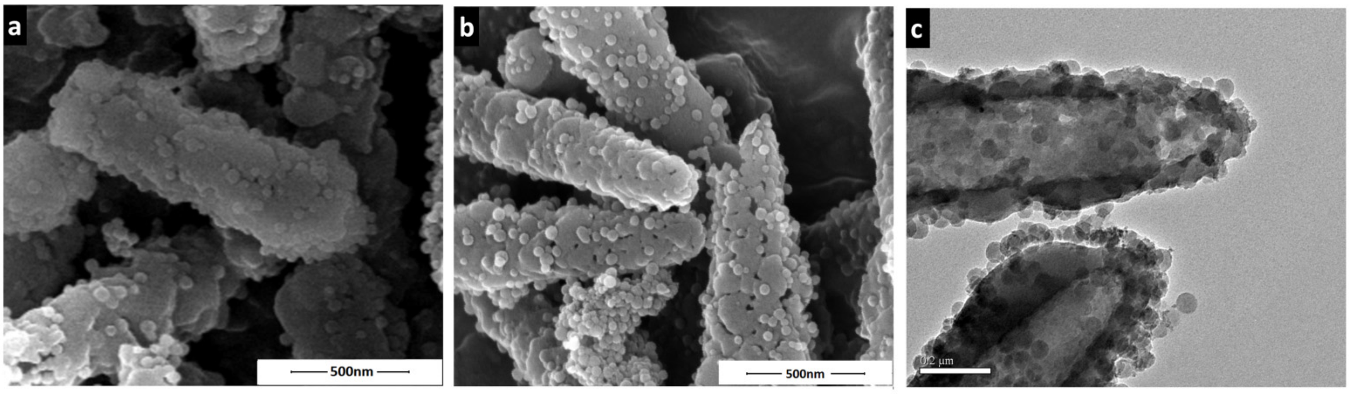

Fig. S4 SEM image of **HMORs-3** (a, 160,000×) and **HMORs-7** (b, 160,000×) and TEM of **HMORs-3**

Table S1 Physical parameters of **m-SiO_2-3**, **HMORs-3**, **m-SiO2-7** and **HMORs-7**

	Length (μm)[a]	Diameter (μm)[a]	Aspect ratio[b, c]
m-SiO_2-3	1.1	0.3	3.4
HMORs-3	1.2	0.5	2.3
m-SiO_2-7	2.0	0.3	7.3
HMORs-7	2.1	0.4	5.6

[a] The average length and diameter were confirmed by SEM (100 particles counted).

[b]Aspect ratio was determined by dividing length by diameter.

[c]The average aspect ratio was calculated by aspect ratio found by each particle.
